# CORRIGENDUM

**DOI:** 10.1111/1759-7714.14468

**Published:** 2022-06-02

In Sun *et a*l.^1^ the following error was published on page 1407.

The data result in Abstract, Results section, was erroneous. The correct data result is ‘IBL clear presentation time (23.59 ± 4.47 vs. 1026.80 ± 318.34 s) (p < 0.001)’ instead of ‘IBL clear presentation time (23.6 ± 4.4 vs. 23.6 ± 4.4 s) (p < 0.01)’. The results section should read as below:

**Results:** An IBL was visible in 98% of patients in the ICGF‐based group, even with low doses of ICG. The ICGF‐based group was significantly associated with a shorter IBL clear presentation time (23.59 ± 4.47 vs. 1026.80 ± 318.34 s) (p < 0.001) and operative time (89.3 ± 31.6 vs. 112.9 ± 33.3 min) (p < 0.01) compared to the MID group. The incidence of postoperative prolonged air leaks was higher in the MID group than in the ICGF‐based group (8/100, 8% vs. 26/98, 26.5%, p = 0.025). There were no significant differences in bleeding volume, chest tube duration, postoperative hospital stays, surgical margin width, and other postoperative complications.

The authors apologize for the error and any inconvenience it may have caused.
